# Upregulation of spinal ASIC1 by miR‐485 mediates enterodynia in adult offspring rats with prenatal maternal stress

**DOI:** 10.1111/cns.13542

**Published:** 2020-12-13

**Authors:** Xue Xu, Yong‐Chang Li, Yan‐Yan Wu, Yu‐Cheng Xu, Rui‐Xia Weng, Cai‐Lin Wang, Ping‐An Zhang, Ying Zhang, Guang‐Yin Xu

**Affiliations:** ^1^ The People's Hospital of Suzhou New District Suzhou China; ^2^ Center for Translational Pain Medicine Institute of Neuroscience Soochow University Suzhou China

**Keywords:** acid‐sensitive ion channel 1, enterodynia, miR‐485, prenatal maternal stress, spinal dorsal horn

## Abstract

**Aims:**

Irritable bowel syndrome (IBS) is a common functional gastrointestinal disease characterized by abdominal pain. Our recent study has shown that the acid‐sensitive ion channel 1 (ASIC1) in dorsal root ganglion (DRG) is involved in stomachache of adult offspring rats subjected with prenatal maternal stress (PMS). MiR‐485 is predicted to target the expression of ASIC1. The aim of the present study was designed to determine whether miR‐485/ASIC1 signaling participates in enterodynia in the spinal dorsal horn of adult offspring rats with PMS.

**Methods:**

Enterodynia was measured by colorectal distension (CRD). Western blotting, qPCR, and in situ hybridization were performed to detect the expression of ASICs and related miRNAs. Spinal synaptic transmission was also recorded by patch clamping.

**Results:**

PMS offspring rats showed that spinal ASIC1 protein expression and synaptic transmission were significantly enhanced. Administration of ASICs antagonist amiloride suppressed the synaptic transmission and enterodynia. Besides, PMS induced a significant reduction in the expression of miR‐485. Upregulating the expression markedly attenuated enterodynia, reversed the increase in ASIC1 protein and synaptic transmission. Furthermore, ASIC1 and miR‐485 were co‐expressed in NeuN‐positive spinal dorsal horn neurons.

**Conclusions:**

Overall, these data suggested that miR‐485 participated in enterodynia in PMS offspring, which is likely mediated by the enhanced ASIC1 activities.

## Graphical abstract

The present study has shown that miR‐485 negatively regulates ASIC1 expression and synaptic transmission in the spinal dorsal horn, thus eventually contributing to enterodynia of PMS offspring. It tips us to prevent the adverse effects on offspring induced by environmental stressors during pregnancy.

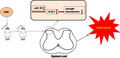

## 1 INTRODUCTION

Irritable bowel syndrome (IBS) is a persistent or intermittent disease characterized by abdominal pain, abdominal distension, and alterations in defecation habits.^1^, ^2^ In particular, the characteristic of enterodynia is more obvious, which has a huge impact on people's lives. Up to now, its specific pathogenesis and effective prevention and treatment options in clinic are still unclear. It is currently believed that the occurrence of IBS is related to gastrointestinal motility abnormalities, intestinal flora imbalance, inflammation, and physical and mental stress in early life.^3^, ^4^, ^5^ Early life stress is mainly divided into two categories: pregnant stress and neonatal stress. Literatures have reported that adverse stressors during pregnancy increase the risk of hypertension, diabetes, depression and anxiety in adult offspring.^6^, ^7^, ^8^ However, the mechanism underlying the pregnancy stress‐induced enterodynia of adult offspring remains largely unclear. Therefore, prenatal maternal stress (PMS) model was used in the present study to further explore mechanisms of early life stress on enterodynia. At present, many studies have been focused on the effects of stressors on the brain areas such as amygdale or the peripheral nervous system such as dorsal root ganglia (DRG) of the offspring.^9^, ^10^ Relatively few studies are focused on the spinal dorsal horn. The spinal cord is the first relay area of sensory information from the periphery to the center. It plays a regulatory role in transmitting and integrating pain information.^11^, ^12^, ^13^ It is, therefore, necessary to explore the role of spinal dorsal horn in enterodynia.

The transmission of biological information mainly depends on the conduction of specific currents of ion channels on the membrane of neurons. Recent studies have also shown that changes in expression and functions of key ion channels and receptors in extrinsic sensory neurons play an important role in the abnormal pain of gastrointestinal diseases.^14^, ^15^, ^16^ Acid‐sensing ion channels (ASICs) are a class of cation channels that are directly activated by extracellular acids and belong to the degenerate protein, epithelial Na^+^ ion channel superfamily (DEG/ENaC), which are widely distributed in central and peripheral nervous systems.^17^ ASICs are divided into at least six isoforms: ASIC1a, ASIC1b, ASIC2a, ASIC2b, ASIC3, and ASIC4. Among them, ASIC1a and ASIC2b are expressed in the spinal cord. ASICs play various roles in pathophysiological processes such as synaptic plasticity, learning and memory, inflammation, and cerebral ischemia.^18^, ^19^, ^20^, ^21^ Our recent study has shown that ASIC1 in DRG plays a positive role in PMS‐induced stomachache.^22^ However, the role of ASIC1 in the spinal dorsal horn on enterodynia remains unclear.

Many miRNAs have been described as regulators in neuropathic pain induced by spinal nerve ligation (SNL) or chronic constriction injury,^23^, ^24^ and some miRNAs are proved to be associated with functional gastrointestinal disorders such as IBS.^25^ However, there is lack of evidence to prove the relationship between miRNAs and chronic enterodynia. Until now, dysregulation of miR‐485 has been reported to be associated with a variety of cancers and regulate tumor cell growth, proliferation, invasion, and migration.^26^, ^27^ In addition, miR‐485 could target ASIC1 expression predicted by bioinformatics software. However, the expression and function of miR‐485 in the enterodynia have not been reported yet. Whether it induces the occurrence of IBS by regulating the expression of ASIC1 remains unknown.

The hypothesis of the present study is that PMS reduced the expression of miR‐485, led to an increase in ASIC1 expression, and enhanced the synaptic transmission of neurons in the spinal dorsal horn, thus contributing to enterodynia of the offspring rats. The expression of ASIC1 and miR‐485 was examined by Western blotting and qPCR. Synaptic transmission and colonic hypersensitivity were also explored. Our data suggest that miR‐485/ASIC1 signaling may be crucial for the development of enterodynia.

## 2 METHODS

### 2.1 Animals and prenatal maternal stress modeling

Specific pathogen‐free (SPF) male and female Sprague Dawley (SD) rats weighing approximately 200 g were provided by the experimental animal center of Soochow University. These rats were housed in the SPF breeding rooms of the animal research center of Soochow University. The light cycle was automatically controlled (12 h of light/dark cycle), and the room temperature was controlled at 22 ± 2°C. The modeling of prenatal maternal stress (PMS) was as described previously.^9^, ^22^ Briefly, starting from the 7th day of pregnancy, the pregnant rats were subjected to one of the following three stressors daily randomly until they delivered: 60 min of water avoidance stress (WAS), 40 min of cold‐restrained stress (CRS), or 20 min of forced swimming stress (FSS). Pregnant control (CON) rats were not handled. To avoid the possible actions of female hormone on pain threshold, only male offspring rats were used in the present study.

### 2.2 Behavioral examination of colorectal distention and drug administration

Colorectal distention (CRD) testing was performed in offspring rats at 6 weeks of age to detect the enterodynia responses in a blinded manner as described previously.^28^, ^29^ For behavioral experiment, 10 μg/μl amiloride (ASIC antagonist, Sigma, St Louis, MO, USA) dissolved in DMSO (Sigma, St Louis, MO, USA) was intrathecally injected in PMS offspring one time or once a day for consecutive 7 days. Patch‐clamp recording was also performed after the last injection of amiloride. In addition, 20 μM miR‐485 agomir or negative control (NC) agomir (GenePharma, Shanghai, China) dissolved in DEPC water was intrathecally injected in PMS offspring one time or once a day for consecutive 7 days for the behavioral or Western blotting experiments.

### 2.3 Western blotting

Western blotting was performed to detect the expression of ASIC1 and ASIC2 in colon‐related segments (T13‐L2) of spinal dorsal horn of CON and PMS offspring rats. The experimental method was followed by our previously published articles.^30^, ^31^ Primary antibodies included rabbit anti‐ASIC1 (1:1000, Alomone Labs, Jerusalem, Israel), rabbit anti‐ASIC2 (1:1000, Alomone Labs, Jerusalem, Israel), and rabbit anti‐GAPDH (1:1000, Goodhere Biotechnology, Hangzhou, China). The secondary antibodies were goat anti‐rabbit peroxidase‐conjugated secondary antibody (1:2000, Multi Sciences Biotech Co., Hangzhou, China).

### 2.4 Quantitative real‐time PCR

Total RNA was extracted from the dorsal horn of spinal cord in CON and PMS offspring rats using TRIzol Reagent (Ambion, Texas, USA). cDNA was synthesized using reverse transcription kit (TransGen Biotech, Beijing, China) following the manufacture's instruction. The primer sequences used in qPCR are shown in Table 1. In addition, the cDNAs of 10 miRNAs and U6 were synthesized using reverse transcription kit (Applied Biosystems, Waltham, MA, USA) following the instruction. The primer sequences used in miRNA qPCR are shown in Table 2.

**Table 1:** The primer sequences used in qPCR

Primers	Sequences (5′ to 3′)
ASIC1‐F	CAGACGTGGAAAGTGCCAGA
ASIC1‐R	GCTCTCGCAGGGATTGTGT
ASIC2‐F	TCCGCCACATCTTCGTGTAT
ASIC2‐R	CCATTGAGGTTGCAGAGGGT
GAPDH‐F	TCTCTTGTGACAAAGTGGACAT
GAPDH‐R	CTCGCTCCTGGAAGATGGTG

**Table 2:** The primer sequences used in miRNA qPCR

Primers	Sequences (5′ to 3′)
miR‐485‐F	CGAGAGGCTGGCCGTGAT
miR‐485‐R	AGTGCAGGGTCCGAGGTATT
mir‐485‐RT	GTCGTATCCAGTGCAGGGTCCGAGGTATTCGCACTGGATACGAATTC
miR‐9a‐5p‐F	GCGCGTCTTTGGTTATCTAGCT
miR‐9a‐5p‐R	AGTGCAGGGTCCGAGGTATT
miR‐9a‐5p‐RT	GTCGTATCCAGTGCAGGGTCCGAGGTATCCGCACTGGATACGACTCATAC
miR‐218a‐5p‐F	GCGCGTTGTGCTTGATCTAA
miR‐218a‐5p‐R	AGTGCAGGGTCCGAGGTATT
miR‐218a‐5p‐RT	GTCGTATCCAGTGCAGGGTCCGAGGTATTCGCACTGGATACGACACATGG
miR‐125a‐5p‐F	AGTGCAGGGTCCGAGGTATT
miR‐125a‐5p‐R	GCGTCCCTGAGACCCTTTAAC
miR‐125a‐5p‐RT	GTCGTATCCAGTGCAGGGTCCGAGGTATTCGCACTGGATACGACTCACAG
miR‐125b‐5p‐F	CGCGTCCCTGAGACCCTAAC
miR‐125b‐5p‐R	AGTGCAGGGTCCGAGGTATT
miR‐125b‐5p‐RT	GTCGTATCCAGTGCAGGGTCCGAGGTATTCGCACTGGATACGACTCACAA
miR‐107‐3p‐F	GCGAGCAGCATTGTACAGGG
miR‐107‐3p‐R	AGTGCAGGGTCCGAGGTATT
miR‐107‐3p‐RT	GTCGTATCCAGTGCAGGGTCCGAGGTATTCGCACTGGATACGACTGATAG
miR‐342‐3p‐F	GCGTCTCACACAGAAATCGC
miR‐342‐3p‐R	AGTGCAGGGTCCGAGGTATT
miR‐342‐3p‐RT	GTCGTATCCAGTGCAGGGTCCGAGGTATTCGCACTGGATACGACACGGGT
miR‐101b‐3p‐F	GCGCGCGTACAGTACTGTGATA
miR‐101b‐3p‐R	AGTGCAGGGTCCGAGGTATT
miR‐101b‐3p‐RT	GTCGTATCCAGTGCAGGGTCCGAGGTATTCGCACTGGATACGACTTCAGC
miR‐101a‐3p‐F	GCGCGCGTACAGTACTGTGATA
miR‐101a‐3p‐R	AGTGCAGGGTCCGAGGTATT
miR‐101a‐3p‐RT	GTCGTATCCAGTGCAGGGTCCGAGGTATTCGCACTGGATACGACTTCAGT
miR‐103‐3p‐F	GCGAGCAGCATTGTACAGGG
miR‐103‐3p‐R	AGTGCAGGGTCCGAGGTATT
miR‐103‐3p‐RT	GTCGTATCCAGTGCAGGGTCCGAGGTATTCGCACTGGATACGACTCATAG
U6‐F	AGAGAAGATTAGCATGGCCCCTG
U6‐R	ATCCAGTGCAGGGTCCGAGG
U6‐RT	GTCGTATCCAGTGCAGGGTCCGAGGTATTCGCACTGGATACGACAAAATA

### 2.5 Immunofluorescence and fluorescence in situ hybridization

The immunofluorescence procedures were according to the article published previously.^32^ The thickness of the spinal cord slice was 14 μm. The primary antibodies incubated on the spinal cord included anti‐ASIC1 (1:50, Alomone Labs, Jerusalem, Israel), anti‐NeuN (1:50, Merck Millipore, Darmstadt, Germany), anti‐GFAP (1:100, Cell Signaling Technology, Danvers, MA, USA), and anti‐CD11b (1:50, Bio‐Rad, California, USA). The secondary antibodies were Alexa Fluor 488 (1:500, Molecular Probes New York) and Alexa Fluor 555 (1:100, Molecular Probes New York). Fluorescence in situ hybridization (FISH) was performed with enhanced sensitive ISH Detection Kit I (POD, Boster MK1030, Wuhan, China). A locked nucleic acid probe with complementarities to miR‐485 was labeled with 5′‐ and 3′‐digoxigenin and synthesized by Exiqon. The detailed method was described in an article published in our laboratory.^25^

### 2.6 Spinal cord slice preparation and patch‐clamp recordings

CON or PMS offspring rats were anesthetized, and an incision was made from the back of rat. After removing the lamina, T13‐L2 spinal cord was exposed and quickly immersed in the pre‐oxygenated (95% O_2_, 5% CO_2_) cold Krebs solution (in mM): 95 NaCl, 1.8 KCl, 1.2 KH_2_PO_4_, 0.5 CaCl_2_, 7 MgSO_4_, 26 NaHCO_3_, 15 glucose, and 50 sucrose, at pH of 7.3–7.4 and an osmolarity of 310–320 mOsm. The soft spinal arachnoid was removed from the surface, then the ventral side of the spinal cord was affixed in a vertical groove, and the agar block was vertically fixed to the center of the vibrating slicer stage with a cyanoacrylate adhesive. Several transverse slices (450 μm thickness) were cut with a Vibratome (Leica, Wetzlar, Germany) while the spinal cord was immersed in cold Krebs solution. The slices were transferred to oxygenated Krebs solution at 31°C for 0.5 h. Recording method of spinal cord patch clamp referred to the previous paper.^33^ Neurons in the lamina II of spinal dorsal horn were selected and recorded using an infrared differential interference contrast (IR‐DIC) video microscope with a 40× magnification objective (Olympus, Japan). All electrophysiological data were collected through a Multiclamp 700B amplifier, Digidata 1440A interface, and ClampEx10.3 software (Molecular Devices, Axon, United States) and filtered at 5 kHz with Bessel filter of amplifier. The patch pipettes were pulled from borosilicate glass (Sutter Instruments, California, USA), using a P‐97 Flaming/Brown micropipette puller (Sutter Instrument, California, USA). Pipette resistance was 5–10 ΜΩ for patch‐clamp recording. Recording external solution was as follows (in mM): 127 NaCl, 1.8 KCl, 1.2 KH_2_PO_4_, 2.4 CaCl_2_, 1.3 MgSO_4_, 26 NaHCO_3_, and 15 glucose, at pH of 7.3–7.4 and osmolarity of 300–310 mOsm. Pipette solution contained (in mM): 140 K‐gluconate, 3 KCl, 10 HEPES, 0.2 EGTA, 4 NaCl, and 2 Ma‐ATP. Spontaneous excitatory post‐synaptic currents (sEPSCs) were recorded as described previously.^33^

### 2.7 Statistical analysis

All values are showed as mean ± standard error of the mean (mean ± *SEM*). Statistical analyses were done using Prism 6 (GraphPad, San Diego, California) software. Before analysis, all data were first tested by normal distribution test. Two‐sample *t*‐test or Mann‐Whitney test was then conducted to determine significance of changes between two groups. One‐way ANOVA and two‐way ANOVA were also performed when it was necessary. *P < 0.05 was considered statistically significant.

## 3 RESULTS

### 3.1 PMS enhanced ASIC1 expression in offspring rats

**Figure 1:**
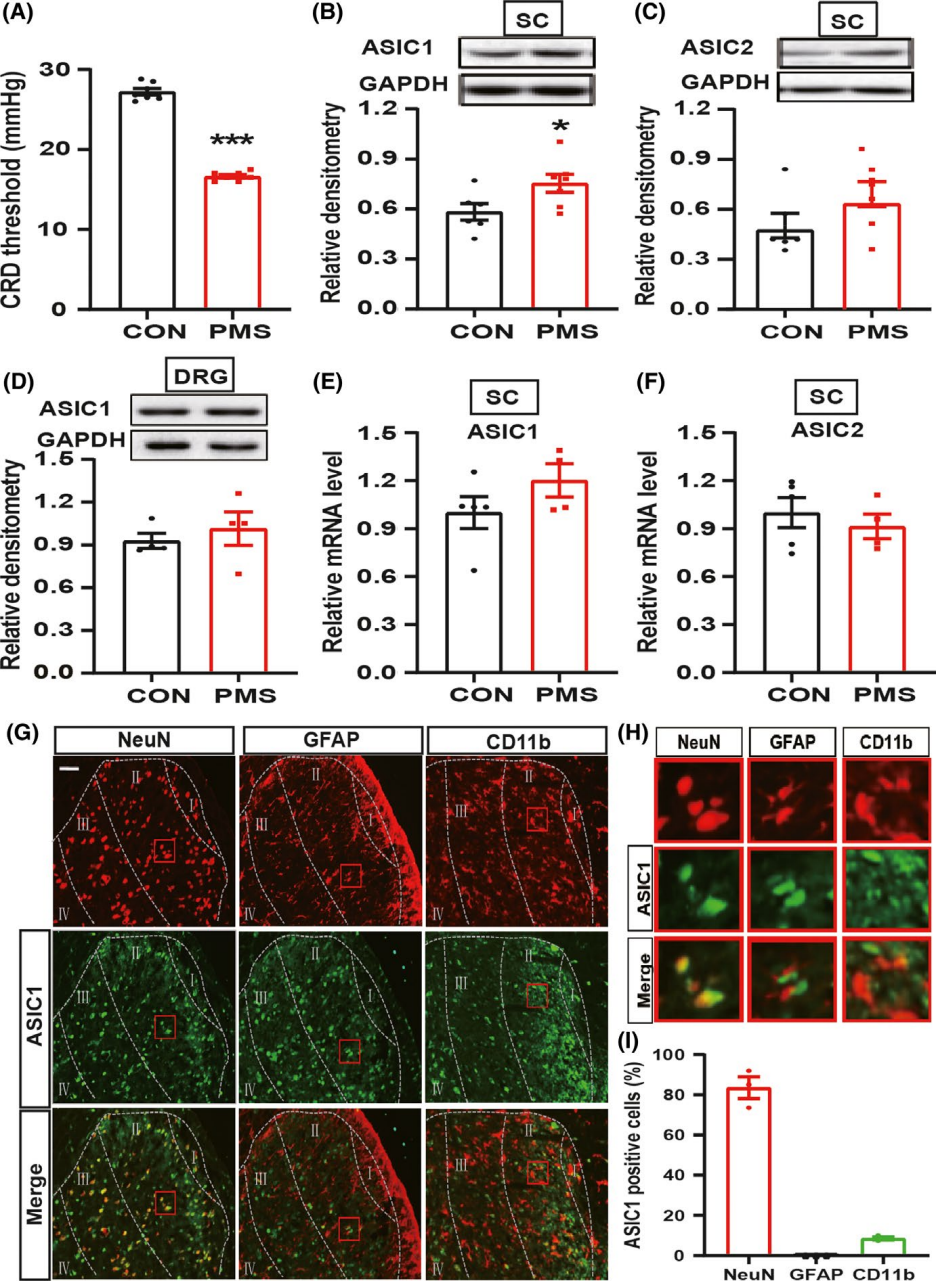
ASIC1, involved in the enterodynia of PMS offspring rats, was co‐localization with spinal dorsal horn neurons. (A) PMS significantly reduced the pain threshold of the offspring rats at the age of 6 weeks compared with CON rats (*n* = 7 for each group, ^***^ *P* = 0.0006 < 0.001 vs. CON, Mann‐Whitney Test). (B) PMS significantly increased ASIC1 expression in T13‐L2 spinal dorsal horn at the age of 6 weeks compared with CON (*n* = 6 for CON group, *n* = 7 for PMS group, ^*^ *P = *0.042 < 0.05 vs. CON, two‐sample *t*‐test). (C) There was no alteration in ASIC2 expression in T13‐L2 spinal dorsal horn at the age of 6 weeks compared with CON (*n* = 6 for CON group, *n* = 7 for PMS group, *P = *0.102 > 0.05 vs. CON, two‐sample *t*‐test). (D) There was no change of ASIC1 expression in T13‐L2 DRG at the age of 6 weeks compared with CON (*n* = 4 for each group, *P = *0.527 > 0.05 vs. CON, two‐sample *t*‐test). (E) There was no alteration in ASIC1 mRNA level in T13‐L2 spinal dorsal horn at the age of 6 weeks compared with CON (*n* = 5 for CON group, *n* = 4 for PMS group, *P = *0.206 > 0.05 vs. CON, two‐sample *t*‐test). (F) There was no change in ASIC2 mRNA level in T13‐L2 spinal dorsal horn at the age of 6 weeks compared with CON (*n* = 5 for CON, *n* = 4 for PMS, *P = *0.516 > 0.05 vs CON, two‐sample *t*‐test). (G) ASIC1 was mainly expressed on NeuN‐labeled neurons, little co‐localized with CD11b, but not GFAP. (H) A partial enlarged view of the co‐standard situation of ASIC1 and three types of cells in spinal dorsal horn. (I) Co‐standard statistics of ASIC1 and three types of cells in spinal dorsal horn

Enterodynia was assessed by the distension threshold (DT) in response to CRD in male offspring at the age of 6 weeks. In consistent with previous reports,^9^, ^34^ PMS significantly reduced the DT (Figure 1A, ^***^ *P* < 0.001, *n* = 7 rats for each group, Mann‐Whitney test), indicating that PMS induced colonic pain in adult offspring. In order to explore whether ASICs were involved in PMS induced the enterodynia, we next examined the expression of ASIC1 and ASIC2 in colon‐related spinal dorsal horn (T13‐L2) of the CON and PMS offspring. The results showed that protein expression of ASIC1 was significantly increased in PMS offspring (Figure 1B, ^*^ *P* < 0.05, *n* = 6 rats for CON and *n* = 7 rats for PMS, two‐sample *t*‐test). However, the protein expression of ASIC2 was not altered (Figure 1C, *P* > 0.05, *n* = 6 rats for CON and *n* = 7 rats for PMS, two‐sample *t*‐test). The protein expression of ASIC1 in T13‐L2 DRGs was not changed (Figure 1D, *P* > 0.05, *n* = 4 for each group, two‐sample *t*‐test). In addition, the mRNA level of ASIC1 in spinal dorsal horn was not altered compared with CON rats (Figure 1E, *P* > 0.05, *n* = 5 rats for CON and *n* = 4 rats for PMS, two‐sample *t*‐test), nor the ASIC2 expression (Figure 1F, *P* > 0.05, *n* = 5 rats for CON and *n* = 4 rats for PMS, two‐sample *t*‐test).

### 3.2 ASIC1 was predominantly expressed in NeuN‐positive cells of spinal dorsal horn

The distribution of ASIC1 in spinal dorsal horn was determined by immunofluorescence staining. As shown in Figure 1, ASIC1 was mainly co‐localized with NeuN, a marker of neurons, not labeled with GFAP‐positive astrocytes and a little co‐localized with microglial marker CD11b (Figure 1G,H). After statistical analysis, the co‐staining rate of ASIC1 out of NeuN‐positive cells was approximately 83.5%, while the ratio of ASIC1 out of CD11b cells was 8.5% (Figure 1I).

### 3.3 PMS enhanced sEPSCs of SG neurons, which was reversed by amiloride

**Figure 2:**
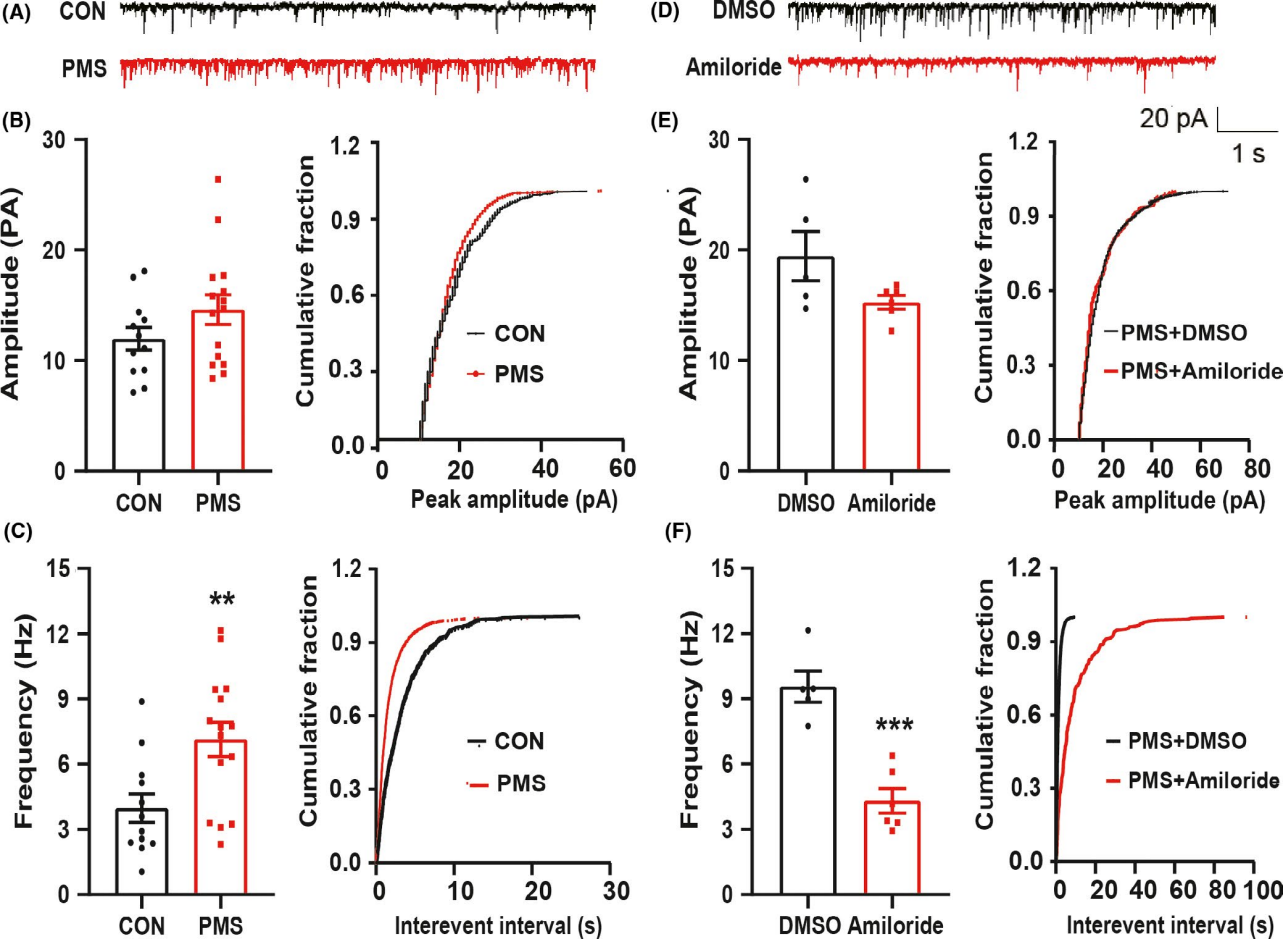
PMS increased sEPSCs in SG neurons which was reversed by amiloride. (A) Representative current traces of sEPSCs recorded in the CON and PMS offspring rats. (B) There was no alteration in amplitude of sEPSCs in T13‐L2 SG neurons at the age of 6 weeks when compared with CON (left, *n* = 12 cells for CON; *n* = 15 cells for PMS, *P = *0.147 > 0.05 vs CON, two‐sample *t*‐test). And cumulative fraction of peak amplitude from one representative SG neuron (right). (C) PMS significantly increased the frequency of sEPSCs in T13‐L2 SG neurons at the age of 6 weeks when compared with CON (left, *n* = 12 cells for CON, *n* = 15 cells for PMS, ^**^ *P = *0.006 < 0.01 vs CON, two‐sample *t*‐test), and cumulative fraction of inter‐event intervals from one representative SG neuron (right). Then, DMSO/amiloride at 10 μg/μl was intrathecally injected for 7 days to perform patch clamp, respectively. (D) Representative current traces of sEPSCs recorded in DMSO and amiloride group rats. (E) There was no alteration in amplitude of sEPSCs in SG neurons compared with DMSO group (left, *n* = 5 cells for DMSO and *n* = 6 cells for amiloride group, *P = *0.080 > 0.05 vs. DMSO, two‐sample *t*‐test), and cumulative fraction of peak amplitude from one representative SG neuron (right). (F) Amiloride significantly decreased the frequency of sEPSCs in T13‐L2 SG neurons compared with DMSO group (left, *n* = 5 cells for DMSO, *n* = 6 cells for amiloride, ^***^ *P = *0.0003 < 0.001 vs. DMSO, two‐sample *t*‐test), and cumulative fraction of inter‐event intervals from one representative SG neuron (right)

The sEPSCs in spinal dorsal horn of CON/PMS offspring were recorded by patch‐clamp techniques. The representative traces from two typical neurons of CON and PMS spinal slices were illustrated in Figure 2A. The frequency of sEPSCs of SG neurons was significantly increased in the PMS group. The frequency of sEPSCs in CON and PMS offspring rats was 3.97 ± 0.65 Hz and 7.13 ± 0.79 Hz, respectively (Figure 2C, left, **P < 0.01, *n* = 12 cells for CON and *n* = 15 cells for PMS, two‐sample *t*‐test). After analysis, the cumulative fraction of amplitude and inter‐event intervals of sEPSCs were shown in Figure 2B,C (right). This suggested that PMS significantly increased the frequency of sEPSCs of SG neurons. However, the amplitudes of sEPSCs in CON offspring rats were 11.96 ± 1.03 pA, while the amplitude of sEPSCs in PMS offspring rats was 14.60 ± 1.34 pA. There was no significant difference between PMS and CON (Figure 2B, left, *P* > 0.05, *n* = 12 cells for CON and *n* = 15 cells for PMS, two‐sample *t*‐test).

Since both the expression of ASIC1 and the spinal synaptic transmission were enhanced in PMS offspring, we assumed that ASIC1 was participated in the increased synaptic transmission. To test th is hypothesis, amiloride, an antagonist of ASICs, was intrathecally injected in 10 μg/μl into the PMS offspring once a day for consecutive 7 days. DMSO was injected as control. The representative traces of sEPSCs from two typical spinal neurons of DMSO‐ and amiloride‐injected rats were illustrated (Figure 2D). The amplitude of sEPSCs of DMSO group and the amiloride group was 19.43 ± 2.22 pA and 15.26 ± 0.61 pA, respectively. There was no significant difference between the two groups (Figure 2E, left, *P* > 0.05, *n* = 5 cells for DMSO and *n* = 6 cells for amiloride, two‐sample *t*‐test). However, the frequency of sEPSCs in DMSO and amiloride was 9.56 ± 0.71 Hz and 4.31 ± 0.57 Hz, respectively. There was a significant reduction in amiloride group (Figure 2F, left, ***P < 0.001, *n* = 5 cells for DMSO and *n* = 6 cells for amiloride, two‐sample *t*‐test). The cumulative fraction of amplitude and inter‐event intervals of sEPSCs was shown in Figure 2E,F (right), suggesting that ASIC1 increased synaptic transmission in the spinal dorsal horn of PMS offspring.

### 3.4 Amiloride relieved enterodynia of PMS offspring

**Figure 3:**
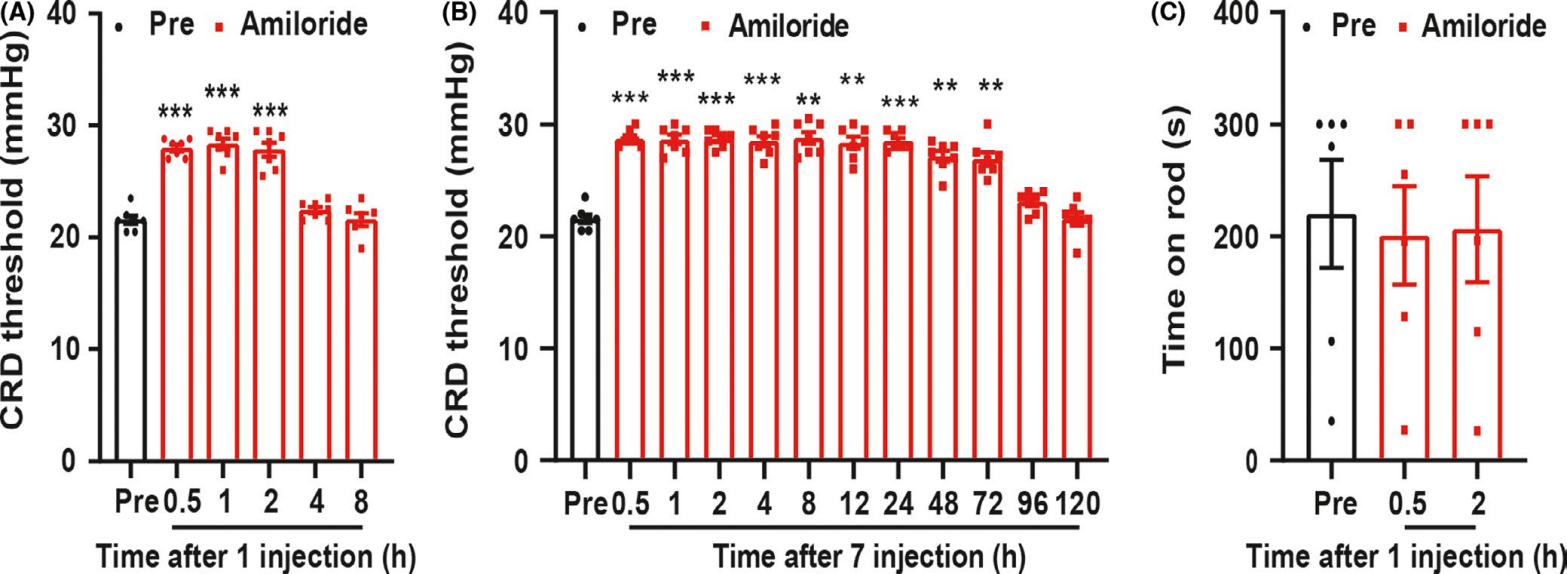
Amiloride attenuated the enterodynia of PMS offspring rats. (A) Time course of effect on visceral pain by intrathecally injecting amiloride for one time. The analgesic effect began at 0.5 h and lasted for 2 h (*n* = 7, ***P < 0.001 vs. Pre, one‐way ANOVA). (B) Time course of effect on visceral pain by intrathecally injecting amiloride for 7 days. The analgesic effect began at 0.5 h and lasted for 72 h (*n* = 7, ^***^ *P* < 0.001 vs. Pre, one‐way ANOVA). (C) Amiloride injection for one time did not affect the time for rats to stay on the rod (*n* = 6, *P* > 0.05 vs. Pre, one‐way ANOVA)

To further determine whether ASIC1 was involved in the enterodynia of PMS offspring rats, 10 μg/μl amiloride was intrathecally injected in PMS offspring rats for one time. After injection of amiloride, CRD threshold was obviously enhanced when compared with Pre, and its effect could last for 2 h (Figure 3A, ***P < 0.001, *n* = 7 rats, one‐way ANOVA). To assess its long‐term analgesia effect, amiloride was injected for 7 consecutive days, and the effect lasted for 72 h (Figure 3B, ^***^ *P* < 0.001, *n* = 7 rats, one‐way ANOVA). In addition, Rota‐rod was performed to detect the effect of amiloride on motor function. There is no effect on the time for PMS offspring rats to stay on the rod (Figure 3C, *P* > 0.05, *n* = 6 rats, one‐way ANOVA). These data indicated that upregulation of ASIC1 expression was involved in PMS‐induced enterodynia.

### 3.5 MiR‐485 was down‐regulated and co‐localized with ASIC1 in spinal dorsal horn of PMS offspring

**Figure 4:**
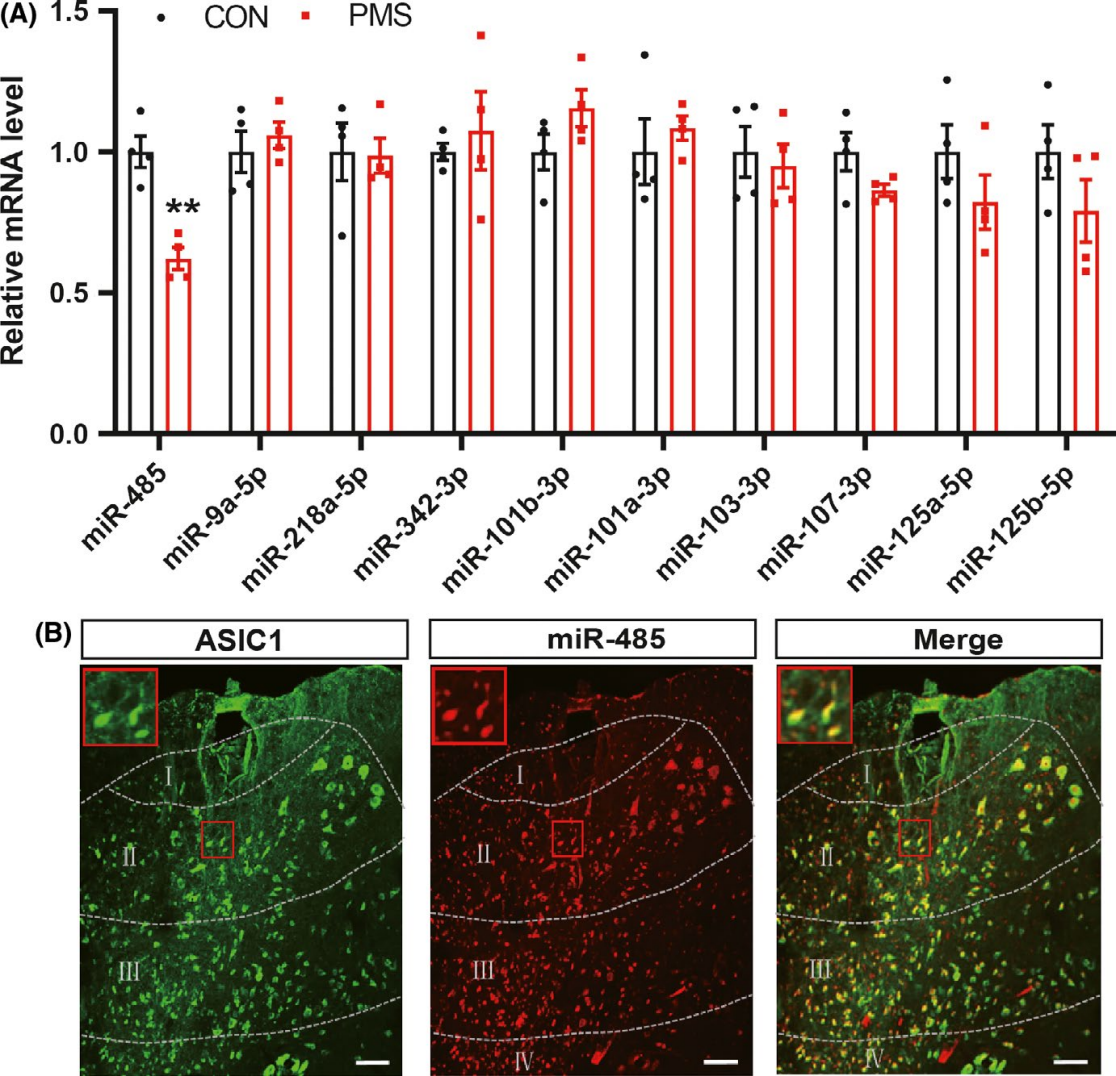
MiR‐485 was reduced in the spinal dorsal horn of PMS offspring rats and co‐expressed with ASIC1 in spinal dorsal horn. (A) PMS significantly reduced miR‐485 expression in T13‐L2 spinal dorsal horn at the age of 6 weeks compared with CON (*n* = 4 for each group, ^**^ *P* < 0.01 vs. CON, two‐sample *t*‐test), while the expression of miR‐9a‐5p, miR‐218a‐5p, miR‐342‐3p, miR‐101b‐3p, miR‐101a‐3p, miR‐103‐3p, miR‐107‐3p, miR‐125a‐5p, and miR‐125b‐5p in PMS offspring rats was not altered compared with CON (*n* = 4 for each group, *P* > 0.05 vs. CON, two‐sample *t*‐test). (B) miR‐485 and ASIC1 were co‐localized in spinal dorsal neurons

Next, we detected whether there were some certain miRNAs, which regulated expression of ASIC1 in the enterodynia induced by PMS. TargetScan software and miRNA.org were used to predicted the upstream miRNAs of ASIC1. According to the bond strength, the expression of 10 miRNAs was detected in the present study. The expression of miR‐485 was significantly decreased in the spinal dorsal horn in PMS offspring compared with CON rats, while the expression of miR‐9a‐5p, miR‐218a‐5p, miR‐342‐3p, miR‐101b‐3p, miR‐101a‐3p, miR‐103‐3p, miR‐107‐3p, miR‐125a‐5p, and miR‐125b‐5p was not altered (Figure 4A, **P < 0.01, *n* = 4 rats for each group, two‐sample *t*‐test). It is known that miRNAs could act on the 3′UTR region of target gene mRNA to inhibit translation. Therefore, to further verify the regulatory relationship between miR‐485 and ASIC1, FISH was carried out. MiR‐485 and ASIC1 were co‐expressed in the same neurons of the spinal dorsal horn (Figure 4B), which provides a basis for the targeted regulation relationship between miR‐485 and ASIC1.

### 3.6 MiR‐485 agomir suppressed ASIC1 expression and synaptic transmission in spinal dorsal horn of PMS offspring

**Figure 5:**
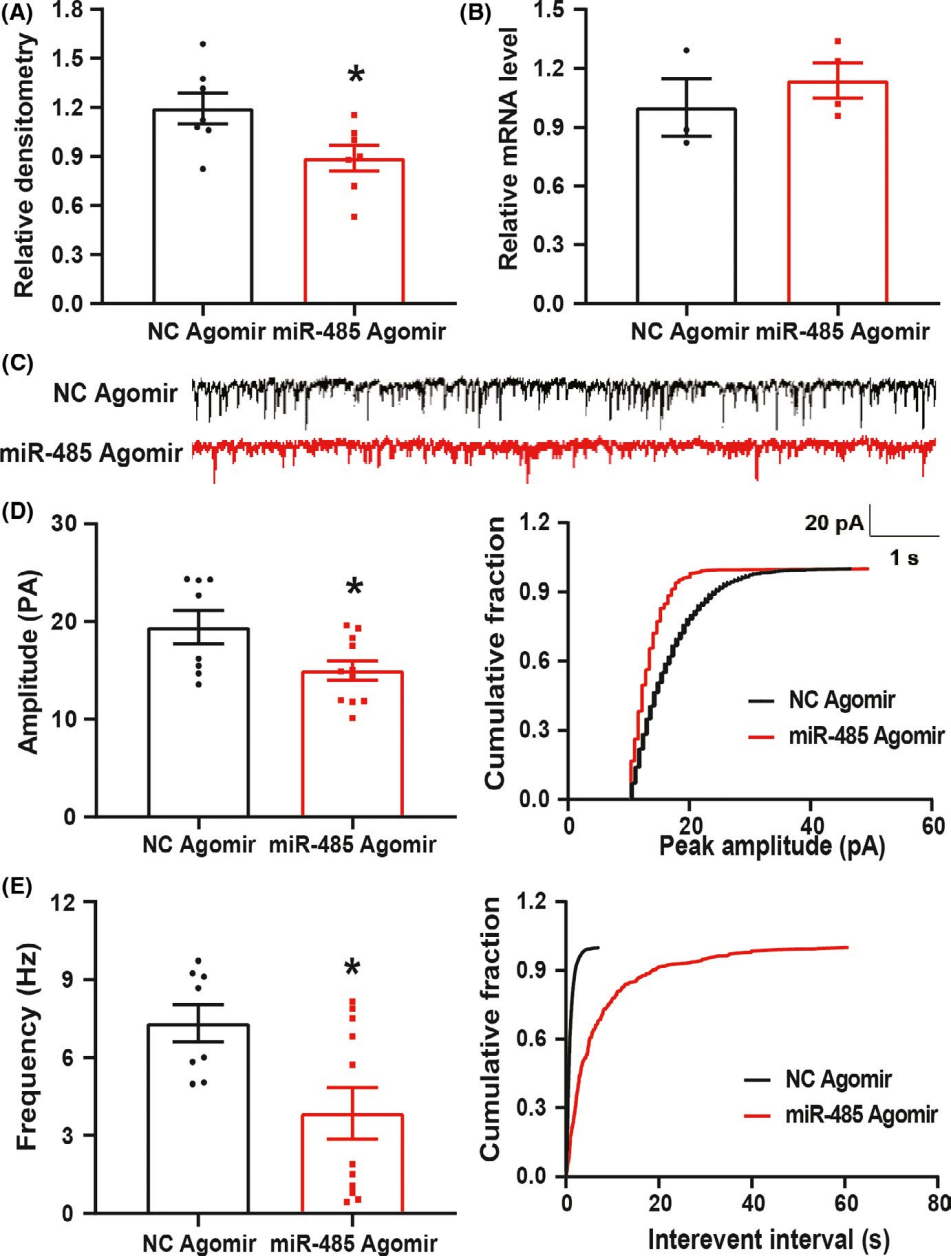
MiR‐485 agomir negatively regulated ASIC1 expression and reversed the synaptic transmission of spinal dorsal horn in PMS offspring rats. (A) The expression of ASIC1 was obviously reduced in spinal dorsal horn after intrathecally injecting miR‐485 agomir for consecutive 7 days (*n* = 7 for each group, *P < 0.05 vs. NC agomir, two‐sample *t*‐test). (B) The mRNA level of ASIC1 was not altered in spinal dorsal horn after intrathecally injecting miR‐485 agomir for once per day for consecutive 7 days (*n* = 3 for NC agomir and *n* = 4 for miR‐485 agomir, *P* > 0.05 vs NC agomir, two‐sample *t*‐test). (C) Representative current traces of sEPSCs recorded after injecting NC agomir and miR‐485 agomir for consecutive 7 days. (D) Compared with NC agomir group, miR‐485 agomir obviously decreased amplitude sEPSCs in SG neurons (left, *n* = 8 cells for NC agomir and *n* = 11 cells for miR‐485 agomir, ^*^ *P* < 0.05 vs. NC agomir, two‐sample *t*‐test), and cumulative fraction of peak amplitude from one representative SG neuron (right). (E) Compared with NC agomir group, miR‐485 agomir obviously decreased frequency of sEPSCs in SG neurons (left, *n* = 8 cells for NC agomir and *n* = 11 cells for miR‐485 agomir, ^*^ *P* < 0.05 vs. NC agomir, two‐sample *t*‐test), and cumulative fraction of inter‐event intervals from one representative SG neuron (right)

We further verified the regulatory relationship between miR‐485 and ASIC1. NC agomir or miR‐485 agomir was intrathecally injected in PMS offspring rats once a day for consecutive 7 days. Western blotting and qPCR were applied to detect the expression of ASIC1. It was shown that miR‐485 agomir significantly reduced the protein expression of ASIC1 in spinal dorsal horn compared with the NC agomir group (Figure 5A, *P < 0.05, *n* = 7 rats for each group, two‐sample *t*‐test). However, the mRNA level was not altered (Figure 5B, *P* > 0.05, *n* = 3 rats for NC agomir and *n* = 4 rats for miR‐485 agomir, two‐sample *t*‐test), indicating that miR‐485 could negatively regulate the expression of ASIC1. Additionally, miR‐485 agomir and NC agomir were intrathecally injected in PMS offspring rats once per day for consecutive 7 days to compare the changes in sEPSCs. The representative traces from two typical neurons of NC agomir and miR‐485 agomir slices illustrated a reduction in both amplitude and frequency of sEPSCs in PMS offspring rats (Figure 5C). The amplitude of sEPSCs in NC agomir group and miR‐485 agomir group was 19.42 ± 1.71 pA and 14.98 ± 1.00 pA, respectively. There was a significant reduction in amplitude in miR‐485 agomir (Figure 5D, left, *P < 0.05, *n* = 8 cells for NC agomir and *n* = 11 cells for miR‐485 agomir, two‐sample *t*‐test). Similarly, the frequency of sEPSCs of NC agomir and miR‐485 agomir was 7.32 ± 0.72 Hz and 3.85 ± 0.99 Hz, respectively. There was a significant reduction in frequency in miR‐485 agomir (Figure 5E, left, *P < 0.05, *n* = 8 cells for NC agomir and *n* = 11 cells for miR‐485 agomir, two‐sample *t*‐test). After data analysis, the cumulative fraction of amplitude and inter‐event intervals of sEPSCs were shown in Figure 5D,E (right). It was indicated that miR‐485 agomir could obviously reduce synaptic transmission in spinal dorsal horn in PMS offspring.

### 3.7 MiR‐485 agomir attenuated enterodynia of PMS offspring

**Figure 6:**
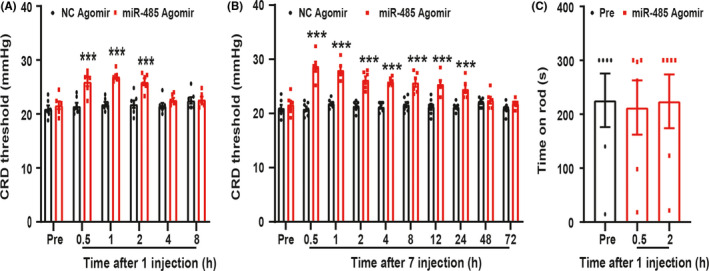
MiR‐485 agomir attenuated the enterodynia of PMS offspring rats. (A) Compared with NC agomir, injecting miR‐485 agomir for one time obviously enhanced the pain threshold of PMS offspring rats (*n* = 7 for NC agomir and *n* = 6 for miR‐485 agomir, ***P < 0.001 vs. NC agomir, two‐way ANOVA). (B) Time course of analgesic effect on visceral pain by intrathecal injection miR‐485 agomir for consecutive 7 days and the effect began at 0.5 h and lasted for 24 h (*n* = 7 for NC agomir and *n* = 6 for miR‐485 agomir, ***P < 0.001 vs. NC agomir, two‐way ANOVA). (C) miR‐485 agomir injection for one time did not affect the time for rats to stay on the rod (*n* = 6, *P* > 0.05 vs. Pre, one‐way ANOVA)

To further verify whether the reduction of miR‐485 is involved in the enterodynia induced by PMS, NC agomir and miR‐485 agomir were intrathecally injected for one time firstly. Compared with NC agomir, miR‐485 agomir obviously enhanced the CRD threshold, and its effect could last for 2 h (Figure 6A, ***P < 0.001, *n* = 7 rats for NC agomir and *n* = 6 rats for miR‐485 agomir, two‐way ANOVA). Next, NC agomir and miR‐485 agomir were intrathecally injected for consecutive 7 days. The analgesia effect of miR‐485 agomir lasted for 24 h (Figure 6B, ***P < 0.001, *n* = 7 rats for NC agomir and *n* = 6 rats for miR‐485 agomir, two‐way ANOVA). In addition, Rota‐rod was performed to detect the effect of miR‐485 agomir on motor function. MiR‐485 agomir had no effect on the time for PMS offspring rats to stay on the rod (Figure 6C, *P* > 0.05, *n* = 6 rats for each group, one‐way ANOVA). These data indicated that the reduction in miR‐485 participated in the enterodynia of PMS offspring.

## 4 DISCUSSION

Chronic enterodynia is considered to be a very unpleasant symptom of IBS patients. It is still not clear about its specific pathogenesis and lacks of effective treatment options in clinical practice. The occurrence of IBS may be related to many factors such as intestinal flora, inflammation, and early life stressors.^35^, ^36^ The present study demonstrated that PMS might be an adverse factor of IBS. The PMS offspring rats exhibited a reduced pain threshold to CRD, which is consistent with the published articles.^9^, ^34^ Importantly, we showed that ASICs in the spinal dorsal horn were participated in the enterodynia in the PMS offspring. Additionally, we provided direct evidence to show that miR‐485 in the spinal dorsal horn regulated the expression and functions of ASICs, thus leading to the development and maintenance of the enterodynia.

Recent studies have shown that the expression of ASIC1 in spinal dorsal horn neurons was significantly upregulated in the inflammatory^37^ and diabetic pain models.^38^ Our previous research has demonstrated that ASIC1 in DRGs was involved in gastric pain of PMS offspring rats.^22^ The present study provided new evidence to support an idea that ASIC1 in spinal dorsal horn mediates the enterodynia of PMS offspring. Although the results of immunofluorescence showed ASIC1 was mainly expressed in neurons of spinal dorsal horn, it is difficult to exclude the roles of ASIC1 expression in the primary sensory nerve endings at the spinal dorsal horn level. It is also difficult to tell whether presynaptic or post‐synaptic site was involved in the spinal synaptic transmission. We showed that frequency of sEPSCs was significantly enhanced while the amplitude was not altered. In addition, the enhanced frequency was obviously reduced after injecting amiloride for 7 days in PMS offspring rats, indicating the role of ASIC1 at the presynaptic site. However, upregulation of miR‐485 by its agomir significantly suppressed both frequency and amplitude of sEPSCs in spinal dorsal horn. This discrepancy may arise from the different interfere approaches. Nevertheless, our data strongly suggest that ASIC1 participates in PMS‐induced enterodynia by enhancing synaptic transmission of spinal dorsal horn neurons. The regulatory pathway may be as follows: Compared with CON group, PMS induced a significant increase in ASIC1 protein expression. This might result in an increased Ca^+^ /Na^+^ influx, hyperexcitation of neurons and promoted synaptic signal transmitting to the brain, eventually leading to enterodynia. Further study had proved that the enhancement of sEPSCs and enterodynia in PMS offspring were reversed by amiloride. Therefore, we concluded that the alteration of synaptic transmission may be related to the enterodynia in PMS offspring.

One potential mechanism by which PMS has a sustained effect on ASIC1 expression and visceral sensitivity is an epigenetic regulation of gene expression.^39^ Epigenetic factors such as DNA methylation, histone acetylation, and miRNAs are all reflections of changes in environmental stimuli.^40^, ^41^ MiRNA, a small RNA sequence, can play a major role in cell differentiation, biological development, and disease development by directly inhibiting target gene translation.^42^, ^43^ Recent years, there were literatures reported the roles of miRNAs in a variety of pregnancy‐related complications such as preeclampsia and FGR, indicating its importance in pregnancy.^44^, ^45^ In general, DNA methylation and histone acetylation are believed to regulate gene expression at mRNA levels while miRNAs are believed to regulate gene expression both at mRNA and protein levels.^46^, ^47^ Since ASIC1 was upregulated only at protein level, it is therefore reasonable to hypothesis that miRNAs participate in PMS‐induced ASIC1 expression at the protein level. To determine which miRNA is involved in the enterodynia during post‐transcriptional regulation of ASIC1 expression, we screened 10 miRNAs obtained from the TargetScan and miRNA.org tools to predict miRNAs upstream of ASIC1. qPCR results showed that only miR‐485 was significantly decreased in spinal dorsal horn of PMS offspring rats within our observations. Importantly, upregulation of miRNA‐485 by agomir not only attenuated the enterodynia but also suppressed the expression of ASIC1 in the spinal dorsal horn. This is, to the best of our knowledge, the first time to demonstrate that miRNA‐485 is a post‐translational regulatory mechanism of ASIC1 expression. This conclusion is based on the following findings. Firstly, the FISH experiment indicated miR‐485 and ASIC1 were co‐expressed in neurons in the dorsal horn of the spinal cord. Secondly, upregulation of miR‐485 by its agomir significantly suppressed the ASIC1 expression and synaptic transmission in spinal dorsal horn. Furthermore, intrathecal injection of miR‐485 agomir markedly relieved the enterodynia of PMS offspring rats, indicating that miR‐485 participated in PMS‐induced chronic colonic pain. Although the detailed interaction machinery needs to be further investigated, our data indicated that miRNA‐485 was involved in the enterodynia of PMS offspring.

## 5 CONCLUSIONS

In summary, the present study has shown that miR‐485 negatively regulates ASIC1 expression and synaptic transmission in the spinal dorsal horn, thus eventually contributing to enterodynia of PMS offspring. These findings shed light on the miR‐485 and ASIC1 signaling pathway in the colonic pain and tip us to prevent the adverse effects on offspring induced by environmental stressors during pregnancy.

## CONFLICT OF INTEREST

No conflicts of interest, financial or otherwise, are declared by the authors.

## Supporting information

Fig S1B

Fig S1C

Fig S1D

Fig S5A

## Data Availability

The data that support the findings of this study are available from the corresponding author upon reasonable request.
