# Supplementary figures and images for: Upregulation of spinal ASIC1 by miR‐485 mediates enterodynia in adult offspring rats with prenatal maternal stress
Source: CNS Neurosci Ther. 2020 Dec 13;27(2):244–55. doi: 10.1111/cns.13542 (PMC7816206; doi:10.1111/cns.13542)

## Full unedited gel/blot for Figure 1 B

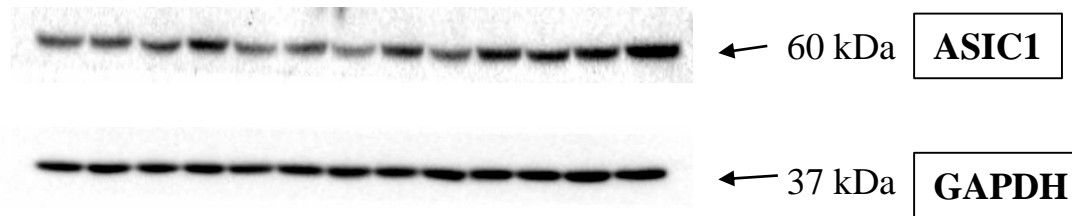

Supplement: Supplementary file 1 — Fig S1B [file CNS-27-244-s001.pdf]

## Full unedited gel/blot for Figure 1 C

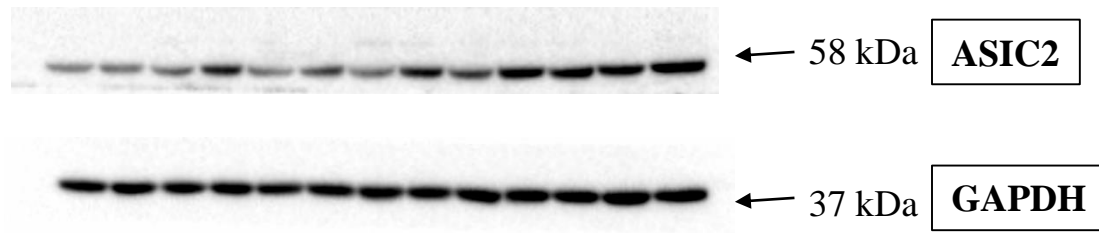

Supplement: Supplementary file 2 — Fig S1C [file CNS-27-244-s002.pdf]

## Full unedited gel/blot for Figure 5 A

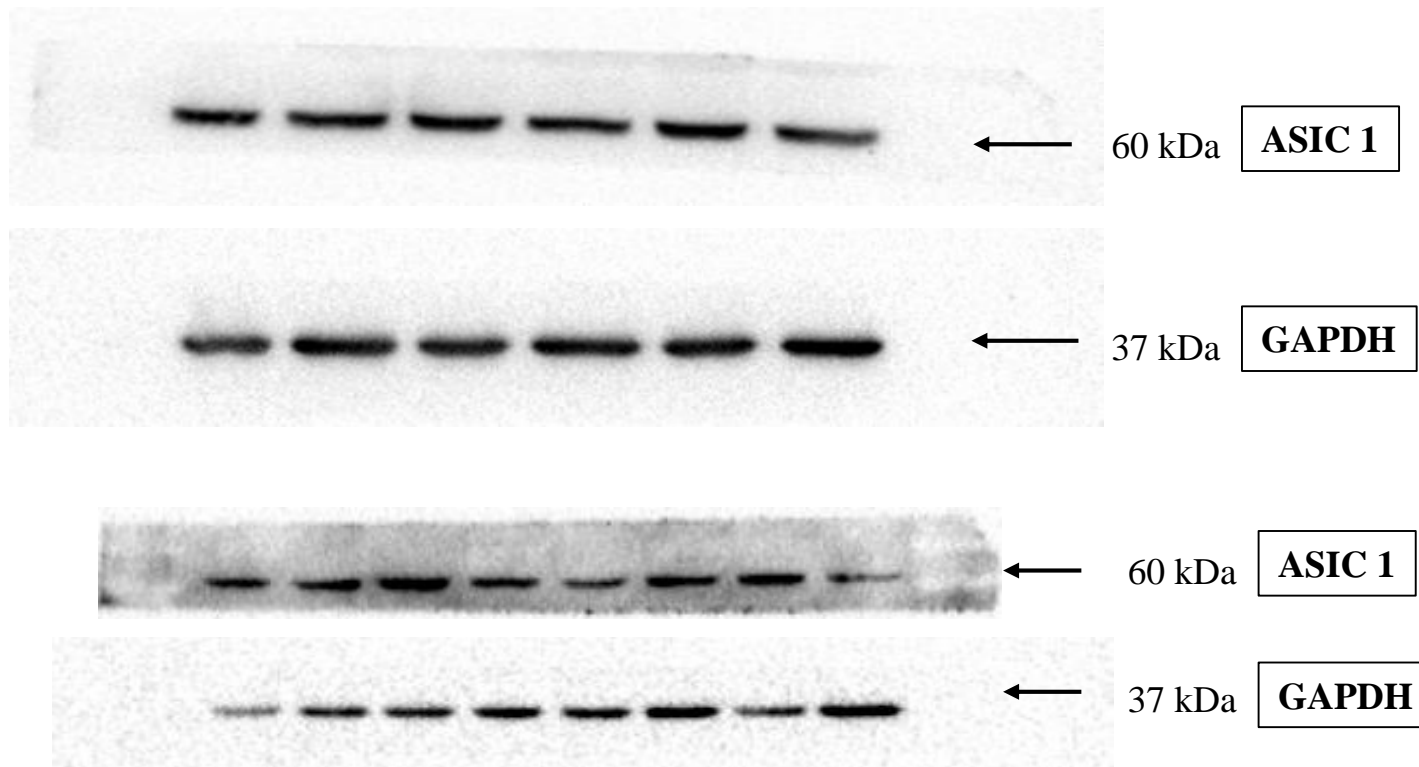

Supplement: Supplementary file 4 — Fig S5A [file CNS-27-244-s004.pdf]
