# Supplementary material for: Upregulation of spinal ASIC1 by miR‐485 mediates enterodynia in adult offspring rats with prenatal maternal stress
Source: CNS Neurosci Ther. 2020 Dec 13;27(2):244–55. doi: 10.1111/cns.13542 (PMC7816206; doi:10.1111/cns.13542)

Full unedited gel/blot for Figure 1 B

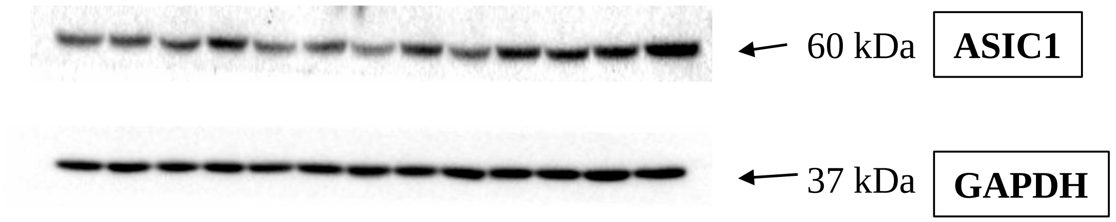

Full unedited gel/blot for Figure 1 C

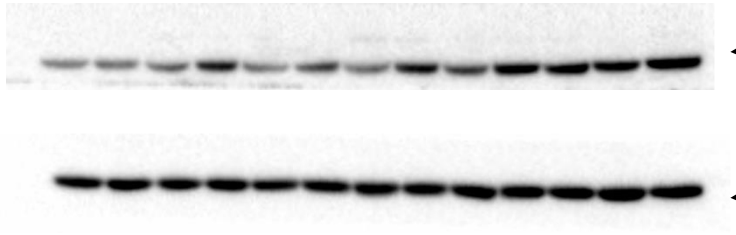

58 kDa **ASIC2**

37 kDa **GAPDH**

Full unedited gel/blot for Figure 1 D

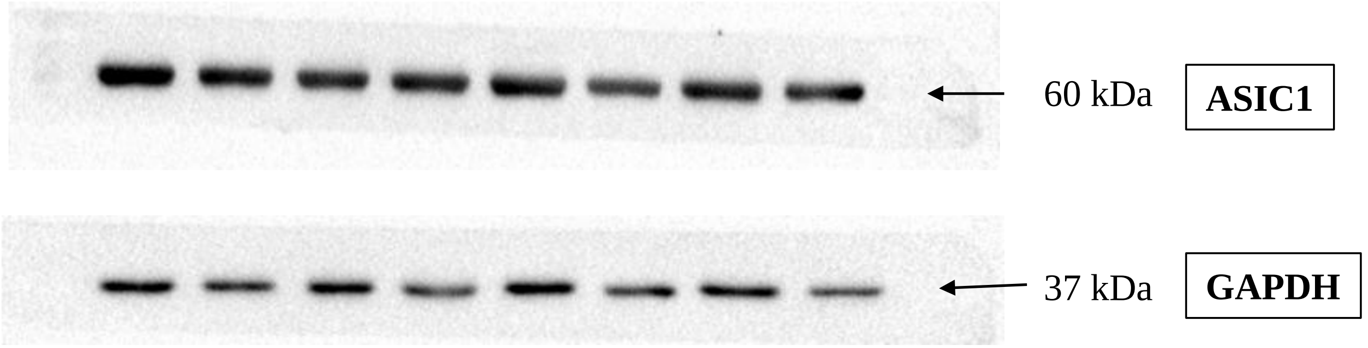

Full unedited gel/blot for Figure 5 A

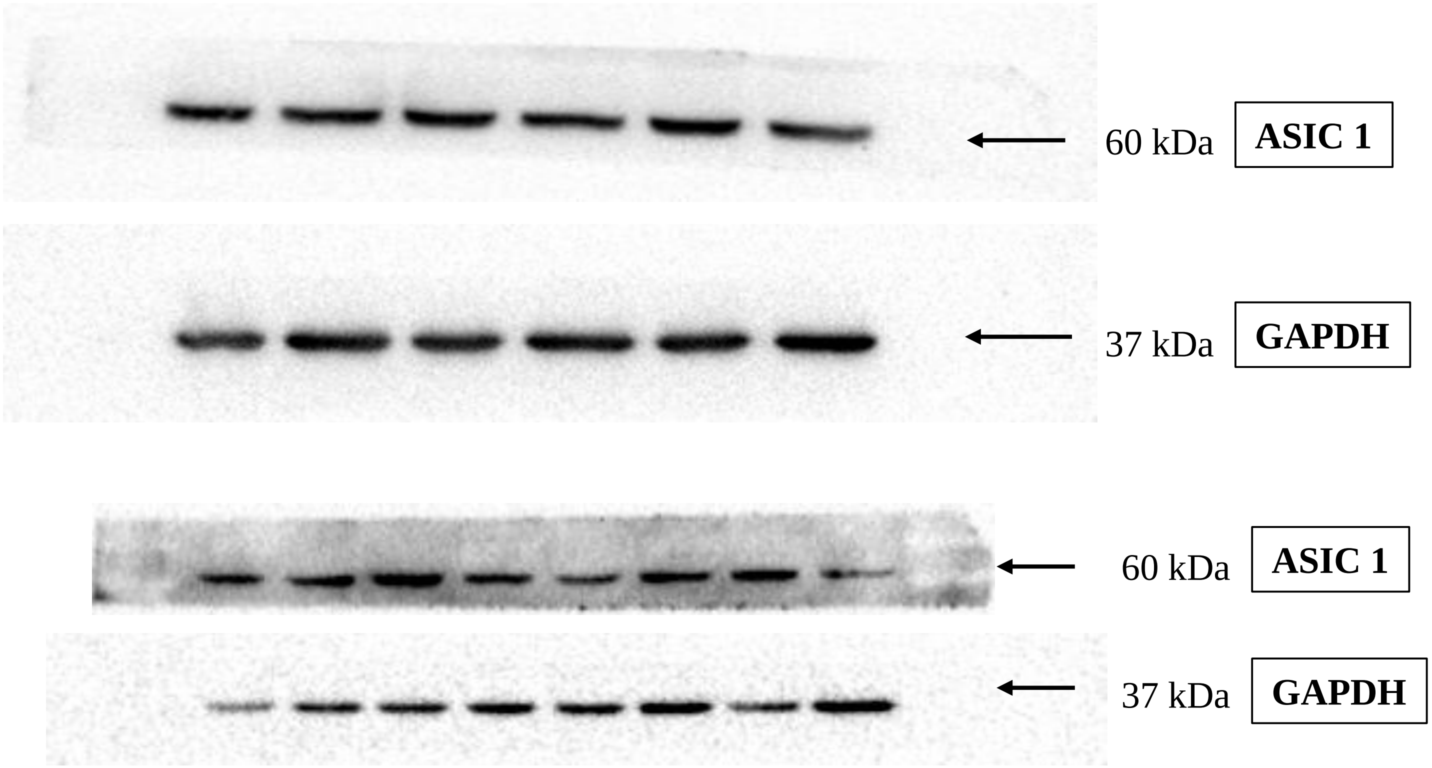

Supplement: Supplementary file 3 — Fig S1D [file CNS-27-244-s003.pdf]
